# Comprehensive Expression Analysis of Rice Armadillo Gene Family During Abiotic Stress and Development

**DOI:** 10.1093/dnares/dst056

**Published:** 2014-01-06

**Authors:** Manisha Sharma, Amarjeet Singh, Alka Shankar, Amita Pandey, Vinay Baranwal, Sanjay Kapoor, Akhilesh K. Tyagi, Girdhar K. Pandey

**Affiliations:** 1 Department of Plant Molecular Biology, University of Delhi South Campus, Benito Juarez Road, Dhaula Kuan, New Delhi 110021, India; 2 National Institute of Plant Genome Research, Aruna Asaf Ali Road, New Delhi 110067, India

**Keywords:** Armadillo, abiotic stress, development, signal transduction

## Abstract

Genes in the Armadillo (ARM)-repeat superfamily encode proteins with a range of developmental and physiological processes in unicellular and multicellular eukaryotes. These 42 amino acid, long tandem repeat-containing proteins have been abundantly recognized in many plant species. Previous studies have confirmed that Armadillo proteins constitute a multigene family in *Arabidopsis*. In this study, we performed a computational analysis in the rice genome (*Oryza sativa* L. subsp. *japonica*), and identified 158 genes of Armadillo superfamily. Phylogenetic study classified them into several arbitrary groups based on a varying number of non-conserved ARM repeats and accessory domain(s) associated with them. An in-depth analysis of gene expression through microarray and Q-PCR revealed a number of ARM proteins expressing differentially in abiotic stresses and developmental conditions, suggesting a potential roles of this superfamily in development and stress signalling. Comparative phylogenetic analysis between *Arabidopsis* and rice Armadillo genes revealed a high degree of evolutionary conservation between the orthologues in two plant species. The non-synonymous and synonymous substitutions per site ratios (*Ka/Ks*) of duplicated gene pairs indicate a purifying selection. This genome-wide identification and expression analysis provides a basis for further functional analysis of Armadillo genes under abiotic stress and reproductive developmental condition in the plant lineage.

## 1. Introduction

Protein repeats are important and common phenomenon shared by all organisms. A repetition in protein sequence allows considerable sequence divergence and multiple binding partners. This observation indicates that the protein repetitions could be an evolutionary link among all members of the proteins.^1^ Unlike domains and motifs, protein repeats can occur individually, even though multiplicity is a known fact for them.^2^ One such evolutionarily conserved protein ensemble is known as Armadillo (ARM) repeat protein family. The occurrence of Armadillo gene family has been reported in proteomes of diverse organisms, such as unicellular eukaryotes (*Chlamydomonas*), non-metazoan (*Dictyostelium*), and recently been reported in higher plants.^3–8^ Armadillo comprises a multigene superfamily, in many plant species. β-Catenin is the vertebrate homologue of Armadillo protein, critical for the development of multicellular organisms.^9,10^ Proteins having ARM repeats are known to participate in multiple cellular processes such as signal transduction, nuclear transport, cell adhesion, and protein degradation.^11–17^ Studies have revealed that ARM proteins have some novel functions in plants, which are determined by their plant-specific functional groups present along with the ARM repeat domain.^14^ This observation indicates the functional significance of these non-described related domains and proteins, and suggests the involvement of many of these ARM repeat proteins in biotic, abiotic, and hormonal signalling networks.^1,13,18^

*Arabidopsis* have been reported with more than 100 ARM repeat proteins, a subset of these has already been characterized.^14^ Studies in *Arabidopsis thaliana*,^7^ *Oryza sativa*,^8^ *Nicotiana tabacum*,^6^ and *Brassica napus*^15^ have helped to categorize ARM proteins into several distinct and specific subgroups on the basis of different functional groups present in conjunction with ARM repeat domain. Unlike animals, several of plant ARM proteins function as components of ubiquitin-proteasome system. There, they act as a part of Plant U-box (PUB) family where the ARM repeat region is preceded by an E3 ubiquitin ligase motif called the U-box^14,15^ thereby targeting a protein for timely degradation, which is a well-conserved mechanism throughout eukaryotes.^3^

Although considerable amount of work has been carried out in characterizing ARM proteins in plants, the functions of the majority of members in this family remain unknown. The objective of the current study is to explore the Armadillo superfamily in rice genome. Using bioinformatics approaches and the information derived from publically available plant databases, the detailed phylogenetic and domain analysis have been carried out for the entire set of ARM proteins. Subsequently, detailed expression analysis during various stages of development and abiotic stress conditions using gene chip microarray and real-time PCR was undertaken to develop a detailed molecular, evolutionary, and functional insights of their possible role *in planta*.

## 2. Materials and methods

### 2.1. Identification of Armadillo superfamily genes in rice genome

Initially, a sequence homology search was performed in RGAP-TIGR 6.1 (http://rice.plantbiology.msu.edu/) using protein sequences of *Arabidopsis* Armadillo proteins as query.^14^ All entries with an *E*-value of ≤1 were considered as the member of ARM family. Subsequently, a protein name (or keyword) search was performed in the NCBI protein database (http://www.ncbi.nlm.nih.gov/protein) to obtain ARM repeats containing sequences from *O. sativa.* All sequences retrieved from these databases were used to perform a pBLAST search again in RGAP6.1. After excluding partial and redundant sequences, all putative ARM repeat proteins were subjected to HHPred scan (http://toolkit.tuebingen.mpg.de/hhpred) for their domain structure.^19^ Sequences that were showing significant homology (*E*-value ≤ 1) with the prototypical ARM repeat proteins such as β-catenin, importin-α, plakophilin, and APC (adenomatous polyposis coli) were considered as members of Armadillo superfamily in *O. sativa*.

The HMM profile for the ARM domain was generated using seed sequences retrieved from Pfam (PF00514) and used to obtain a consensus model using HMMER package 3.0.^20^ The HMM profile was used as query to search all the annotated proteins in whole rice genome at TIGR (*O. sativa*; TIGR release 6.1; ARM domain proteins). In addition, 141 putative protein sequences of ARM superfamily available in InterPro database (http://www.ebi.ac.uk/Tools/pfa/iprscan) were retrieved and further scanned using HMM_ARM signature matrix as well as HHPred (http://toolkit.tuebingen.mpg.de/hhpred) for the presence of tandem ARM repeats. Finally, the deduced protein sequences of predicted Armadillo genes obtained from all the above searches were analysed for any additional motifs or domains using SMART (http://smart.embl-heidelberg.de) and Pfam (http://pfam.sanger.ac.uk/) databases. Various properties and attributes of ARM genes such as locus ID, protein length (AA), tBLASTx match with *Arabidopsis*, and expression evidence in terms of total number of ESTs were extracted from RGAP 6.1 and full-length cDNA accession from KOME (http://cdna01.dna.affrc.go.jp/cDNA) and NCBI (http://www.ncbi.nlm.nih.gov). The HMM search was also performed in the recently released version 7.0 of rice pseudo-molecule database, and the number of predicted Armadillo genes was found to be the same as in version 6.1.

### 2.2. Phylogenetic analysis

To investigate the evolutionary relationship of Armadillo genes in rice, phylogenetic analysis was carried out using the full-length protein sequences of rice and *Arabidopsis*^14^ ARM repeat proteins. In addition, to investigate evolutionary relationships within the ARM/U-box gene families of both the species, a combined phylogenetic tree was generated using complete protein sequences. These phylogenetic trees were constructed by neighbour-joining algorithm employing MEGA 5.0.^21^ The bootstrap analysis was performed using 1000 replicates and the branch lengths corresponded to phylogenetic distances are in units of the number of amino acid substitutions per site.

### 2.3. Nomenclature, chromosomal distribution, and duplication of rice Armadillo genes

The genes were uniformly named in numerical order starting from *OsARM1* to *OsARM109*. To avoid multiple designations being applied to the same gene, previous nomenclature has been kept unchanged (Supple-mentary Table S1). The location of rice Armadillo genes was determined using chromosomal map tool available as Oryzabase-Integrated Science Database (http://viewer.shigen.info/oryzavw/maptool/MapTool.do). The duplication of Armadillo genes on segmentally duplicated regions was determined using ‘Paralogons in *Arabidopsis*’ (http://oldwolfe.gen.tcd.ie/athal/all_results) and segmental genome duplication database for rice (http://rice.plantbiology.msu.edu/segmental_dup/100kb/segdup_100kbshtml) at a maximum length distance permitted between collinear gene pairs of 100 kb. For finding tandemly duplicated candidates, genes with separation by five or less genes were selected.

### 2.4. Estimating age of duplicated paralogue gene pairs

To calculate the age of segmentally duplicated Armadillo paralogues, the pairwise alignment of Armadillo gene pairs from *Arabidopsis* and rice was performed using Clustal X 2.0.12. The age of a duplication event was estimated by the number of synonymous substitution per synonymous site (*Ks*). The *Ks* values of the duplicate Armadillo gene pairs were estimated by the program K Estimator 6.1.^22^ Based on synonymous substitutions per year (*λ*) of 1.5 × 10^−8^ for *Arabidopsis*,^23^ 6.5 × 10^−9^ for rice,^24^ and by substituting the calculated *Ks* values, the approximate age of duplicated events of the duplicate Armadillo gene pairs was estimated (*T* = *Ks/2λ*). The selection pressure for these duplicate Armadillo paralogue gene pairs was calculated as *Ka/Ks* ratio.

### 2.5. Plant material, growth conditions, and stress treatment

The tissue was harvested from field-grown rice plants (*O. sativa* ssp. *Indica* var. IR64) at different stages of panicle and seed development.^25^ For the stress treatment, sterilized IR64 rice seeds were grown in culture room conditions at 28 ± 1°C with a daily photoperiodic cycle of 14 h light and 10 h dark. After 7 days growth, different stress treatments were subjected to the seedlings. Salt treatment was given by transferring the seedlings into 200 mM NaCl solution; for cold treatment, seedlings were kept at 4 ± 1°C in sterile water; and for dehydration, seedlings were air-dried on a Whatmann sheet at 28 ± 1°C for 3 h. Parallel control samples were prepared by keeping the seedlings in sterile water for 3 h. Treated seedlings were immediately frozen in liquid nitrogen.

### 2.6. Microarray-based gene expression analysis

Genome-wide microarray analysis was performed according to Ray *et al.*^25^ to generate the expression profile of *OsARM* genes. The samples for microarray experiment included 3 vegetative stages (mature leaf, 7-day-old seedling, and their roots), 11 reproductive stages (P1–P6 and S1–S5; representing panicle and seed developmental stages, respectively), and 3 abiotic stress conditions, i.e. cold, salt, and dehydration. Total RNA was isolated from three biological replicates for each stage/treated tissue and microarray experiments were carried out using 51 Affymetrix Gene Chip Rice Genome Arrays (Gene Expression Omnibus, GEO, platform accession number GPL2025) as described in Ray *et al.* The raw data (excel) files generated from all the chips were imported to Array Assist 5.0 software (Stratagene, La Jolla, CA, USA) for detailed analysis. Microarray expression data have been deposited in the gene expression omnibus (GEO) at NCBI under the series accession numbers GSE6893 and GSE6901 by Ray *et al.*^25^ The downstream analysis was performed according to Ray *et al.*^25^

### 2.7. Expression analysis by MPSS

The expression profile for those genes, which could not be represented on Affymetrix rice Gene Chip^®^, was generated from rice MPSS (Massively Parallel Signature Sequence) database (http://mpss.udel.edu/rice/). Analysis was performed using five different selected libraries to retrieve 17 bp signature sequences. Only those signatures were included in the analysis, which were unique to the genome and were transcribed from the respective strand of the gene (Classes 1 and 2). A TPM (transcript abundance values per million) cut-off of ≥3 was set to avoid the background signal. The normalized TPM were used to assess the expression profile.

### 2.8. Quantitative expression analysis by real-time PCR

Real-time PCR was performed to verify the microarray expression pattern for a few selected *OsARM* genes, expressed significantly during three abiotic stresses and in various rice tissues/developmental stages. Primers were made for all the selected genes preferentially, from the 3′ end, employing PRIMER EXPRESS (Applied Biosystems, USA), with default settings. Each primer was checked using BLAST tool of NCBI for its specificity for the respective gene, and also was confirmed by the dissociation curve analysis after the PCR reaction. Four micrograms of DNase-treated total RNA were used to synthesize the first-strand cDNA in 100 μl of reaction volume using high-capacity cDNA Archive kit (Applied Biosystems). KAPA SYBR FAST master mix (KAPA BIOSYSTEMS, USA) was used to determine the expression levels for the genes in ABI Prism 7000 Sequence detection System (Applied Biosystems). Two biological replicates of each sample were used for the real-time PCR analysis. The average of three technical replicates for each sample was calculated to obtain the Ct value. To normalize the variance among samples, actin was used as the endogenous control. Relative expression values were calculated by employing the ΔΔCt method and normalized the data against the maximum average expression value from microarray. The Pearson correlation coefficient (*r* value) between microarray and real-time PCR was calculated. The (*r*)-values range between −1 and +1; positive *r*-values correspond to correlated data sets, negative values to anti-correlated data sets, and values close to zero to non-correlated. To calculate the real-time PCR efficiency, serial dilutions of 10 folds were made of selected cDNA samples. Target assay was performed for ACTIN gene. Using the standard curve plotted for the Ct value and the log copy number (Supplementary Fig. S1), the slope of the line was calculated. Using the formula (Efficiency = 10^(−1/Slope)^−1), efficiency was calculated.

### 2.9. Promoter analysis

To find the various *cis*-acting regulatory elements in the promoter of prospective stress-inducible rice Armadillo genes, 1 kb upstream sequence from translation start site was extracted from RGAP 6.1. The upstream sequence was subsequently scanned in PlantCARE^26^ database for the presence of various *cis*-regulatory elements. Various motifs involved in stress responsiveness and development were identified and their positions were marked.

## 3. Results

### 3.1. Identification of Armadillo superfamily in the rice genome

In order to identify and categorize Armadillo protein family in rice on a genome-wide level, multiple approaches were employed. Together, BLASTp and HMM profile search resulted into a list of 133 *OsARM* genes. In addition, 141 protein sequences from InterPro database (http://www.ebi.ac.uk/interpro/) and 1055 sequences from NCBI of Armadillo superfamily in rice were retrieved. Although ARM repeat proteins do not essentially share a large sequence similarity, they do share a related structure.^27^ Taking into account that true repeat proteins contain multiple repeats and detection of all these repeats by domain prediction servers is not feasible because they often are not exact enough to identify certain motifs varying in terms of sequence and number of repeat units.^28^ Henceforth, all the combined hits obtained from above searches were subsequently analysed in the HHpred server (http://toolkit.tuebingen.mpg.de/hhpred).^19^ HHpred provide highly sensitive detection of HEAT/ARM repeats by implementing pairwise comparison of hidden Markov models (HMMs) profile and for structure prediction with a low false-positive rate.^19^ Following the intensive database search, HMM search, structure, and domain analysis, 158 Armadillo genes (*E*-value ≤ 1 × 10^−10^) were identified in the rice genome (Supplementary Table S1).

### 3.2. Phylogenetic and domain analysis of OsARM repeat proteins

**Figure 1.**
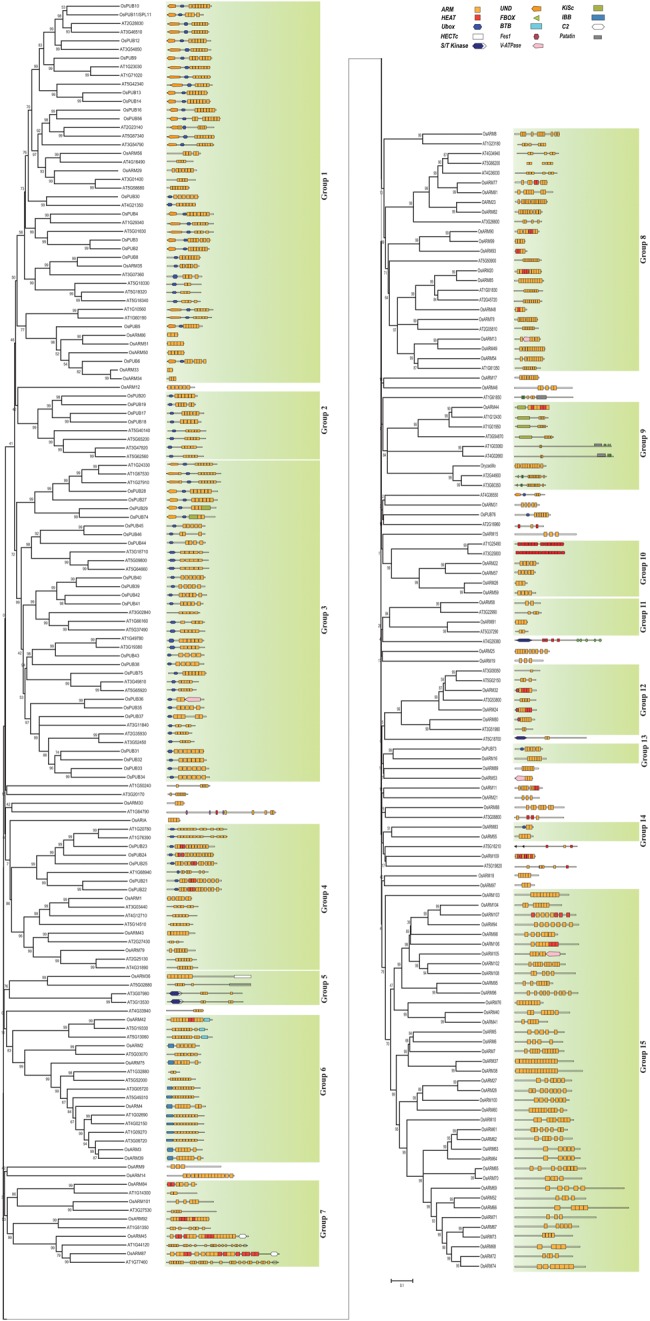
Phylogenetic relationship between *Arabidopsis* and rice *ARM* superfamily genes. A phylogeny created with the full-length protein sequences of ARMs in *Arabidopsis* and *O. sativa*. The alignment of ARM repeat protein sequences of rice Armadillo genes was done using ClustalX 2.0.12. An unrooted neighbour-joining (NJ) tree was generated using the *p*-distance substitution model in MEGA 5. Bootstrap analysis was performed with 1000 replicates to obtain a support value for each branch. Based on bootstrap score, the phylogenetic tree is divided into 15 groups. The domain organization and number and position of ARM repeats in rice have been predicted by the sequence analysis in HHpred and Pfam. The domain organization of *Arabidopsis* ARM proteins has been adapted from Mudgil *et al.* 2004. The representative domain organization of each gene is shown on the right.

To elucidate the evolutionary significance of ARM proteins across species, comparative phylogenetic analysis was performed using complete protein sequences of OsARMs and their respective *Arabidopsis* orthologues from previous study by Mudgil *et al.*^14^ The result showed that rice ARM repeat proteins coincide with specific ARM gene subtypes in *Arabidopsis*, signifying that ARM repeats might have co-evolved between the monocots and eudicots (Fig. 1). Additionally, several other accessory functional domains were found to be present in all predicted ARM repeat proteins. As observed in *Arabidopsis*, the U-box containing ARM repeat proteins (OsPUB-ARM) were found to outline a major group with 47 of 158 ARM repeats proteins in the rice genome. The unique UND region was also recognized based on sequence homology with the *Arabidopsis* UND/U-box proteins. Interestingly, the number of UND/U-box proteins was found to be equivalent (17) in both rice and *Arabidopsis*. Moreover, we observed that rice ARM proteins also display similar organization of ARM repeats and other domains when compared with well-characterized orthologues of *Arabidopsis* ARM family. Other known domains found in rice are five Importin-α proteins with Importin-β-binding (IBB) domain followed by five to seven ARM repeats. A large number of additional motifs were found to be associated within the predicted set of ARM proteins. These included domains implicated in protein–protein interactions such as HEAT and BTB. Again, several domains recognized for their role in protein ubiquitination such as U-box (47), HECT (1), Fes1 (3), and F-box (1) were found in conjunction with ARM domain in rice. In addition, two calcium-dependent phospholipid binding C2 domain responsible for membrane targeting and three microtubule-associated kinesin motor domain proteins were also found to be present. The protein sequence of sole orthologue of Arabidillo-1, 2 in rice (LOC_Os10g41360) derived from RGAP (http://rice.plantbiology.msu.edu) did not contain NLS/F-box N-terminal; however, a BAC clone search (GenBank Accession: AAG60190) in NCBI has confirmed the similar domain organization.

### 3.3. Chromosomal distribution and duplication of Armadillo genes in rice

**Figure 2.**
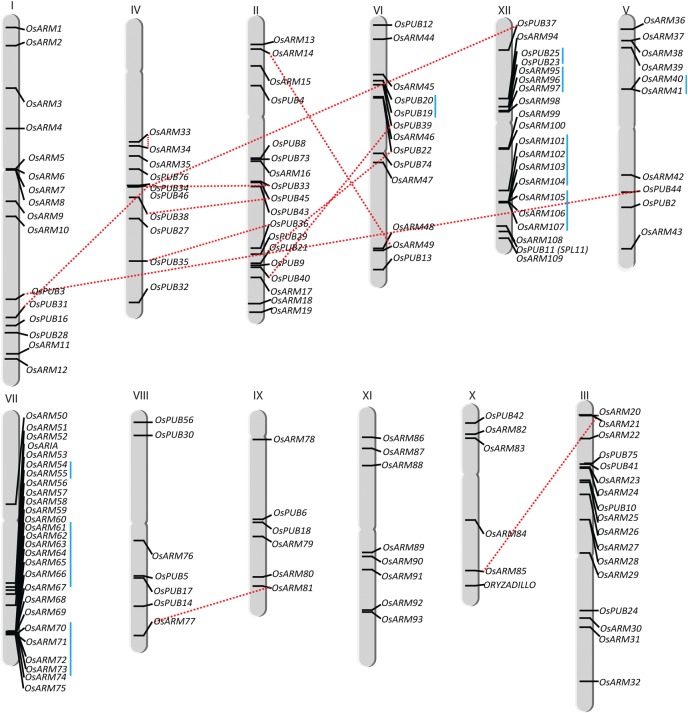
Chromosomal localization of *OsARM* superfamily genes on 12 chromosomes of rice. *OsARMs* have been mapped by their positions on 12 chromosomes. Respective chromosome numbers are written at the top. Red lines join the genes, present on duplicated segments of the genome. Tandemly duplicated genes are shown with the blue vertical lines.

To determine the chromosomal distribution of Armadillo genes in rice, chromosomal maps were constructed. *ARM* genes were found to be variably distributed on all chromosomes with maximum 27 genes on chromosome 7 and minimum 6 on chromosome numbers 9 and 10. This is followed by chromosome numbers 12, 2, 3, and 1 with 20, 18, 17, and 16 genes, respectively (Fig. 2). Sixteen pairs of *OsARM* genes were found to be segmentally duplicated (Supplementary Table S2). Moreover, tandem duplication was observed among 30 genes forming 9 groups on chromosome numbers 5, 6, 7, and 12. Maximum tandemly duplicated genes were found on chromosome numbers 7 and 12 with four and three groups of genes. Whereas, in *Arabidopsis*, 13 pairs of ARM genes were found to be segmentally duplicated and 10 genes with four groups were found to be exhibiting tandem duplication (Supplementary Table S3). Predictably, all segmental as well as tandemly duplicated genes share similar domain organization and almost same number of ARM repeats. We further estimated the approximate age of segmentally duplicated Armadillo paralogous gene pairs from *Arabidopsis* and rice (Supplementary Table S4). The number of synonymous substitutions per synonymous site (*Ks*) is generally used to estimate the evolutionary age of duplicate gene pairs.^29^ The nucleotide sequence of three duplicated gene pairs, *At1g20780–At1g76390*, *At3g06210–At5g18980*, and *At3g01450–At5g14790* from *Arabidopsis* showed a *Ks* value of 0.673, 0.622, and 0.615, respectively, indicating that their duplication might have occurred 22, 20.7, and 20.4, MYA (million years ago) consistent with its divergence from the genus *Brassica∼*12–20 MYA,^30^ but four of the gene pairs *At1g01830–At2g45720*, *At1g23030–At1g71020*, *At5g09800–At5g64660*, and *At3g49810–At5g65920* showed a *Ks* value of 1.656,0.787, 0.715, and 0.719, respectively, indicating that their duplication might have occurred 55, 26, 23.83, and 23.9 MYA before the appearance of crucifers ∼24–40 MYA.^31^ Likewise, the segmentally duplicated pair *OsARM13-OsARM49* showed a *Ks* value of 1.15, signifying that its duplication might have occurred ∼88.65 MYA, subsequent to the divergence between monocots and dicots (100–200 MYA), while six other gene pairs *OsPUB31–OsPUB34*, *OsPUB2–OsPUB3*, *OsARM20–OsARM85*, *OsPUB35–OsPUB36*, *OsPUB21–OsPUB22*, and *OsARM77–OsARM81* showed a *Ks* value of 0.6, 0.54, 0.47, 0.49, 0.81, and 0.68 representing duplication at ∼46.45, 41.75, 35.98, 37.49, 62.56, and 52.51 MYA after the divergence of poaceae from the common ancestor ∼55–70 MYA.^32^ Interestingly, two of the segmentally duplicated pair in *Arabidopsis* (*At1g22870–At1g71410* and *At2g25130–At4g31890*) and one in rice (*OsARM33–OsARM34*) with corresponding *Ks* values of 0.793, 0.536, and 0.06 present an evidence for a recent duplication at 1.7, 2.6, and 0.46 MYA.

### 3.4. Expression analysis of OsARM genes under abiotic stress conditions

**Figure 3.**
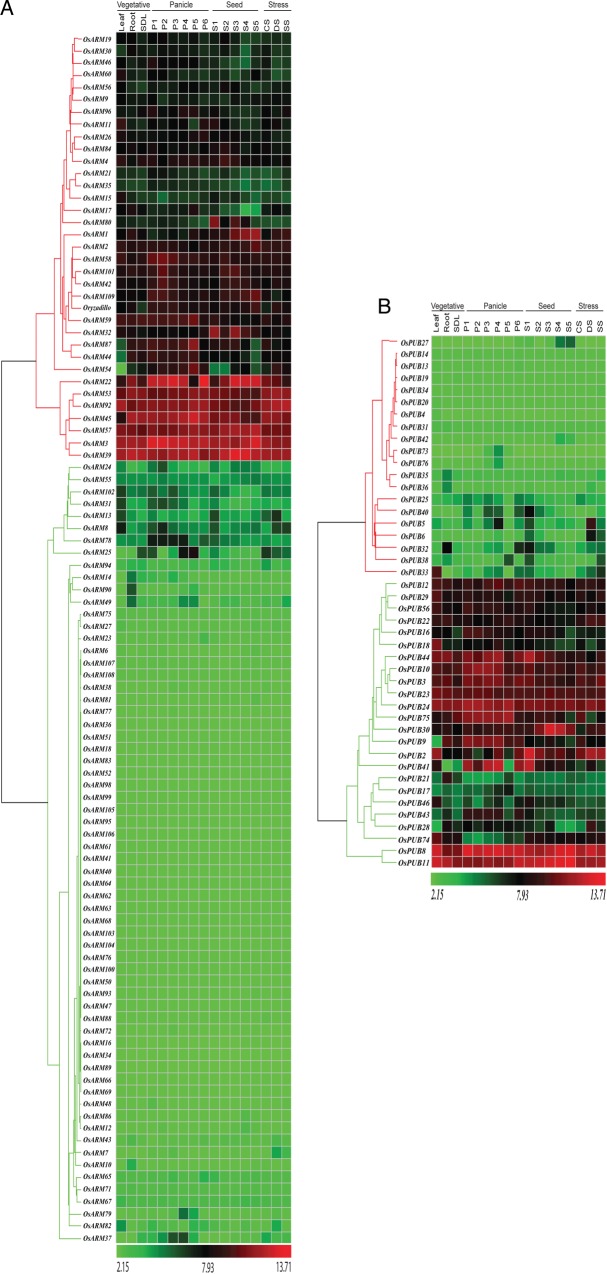
Expression profiles of *OsARM* superfamily genes. Reproductive development comprising six stages of panicle [P1 (0–3 cm), P2 (3–5 cm), P3 (5–10 cm), P4 (10–15 cm), P5 (15–22 cm), and P6 (22–30 cm)] and five stages of seed [S1 (0–2 DAP), S2 (3–4 DAP), S3 (4–10 DAP), S4 (11–20 DAP), and S5 (21–29 DAP)] development. Clustering of the expression profile was done with log-transformed average values taking mature leaf as baseline. Three experimental stress conditions are denoted as CS, cold stress; DS, drought stress; SS, salt stress; and S, control, 7-day-old unstressed seedling. A gene is considered differentially expressed during reproductive development if up- or down-regulated at least 2-fold, with respect to three vegetative controls (mature leaf, root, and 7-day-old seedling) and with respect to 7-day-old unstressed seedling in the case of abiotic stress. The colour scale at the bottom of the heat map is given in log2 intensity value. (A) The expression profile for *OsARM* genes other than U-box. (B) Expression profile of *OsPUB/ARM* genes.

**Figure 4.**
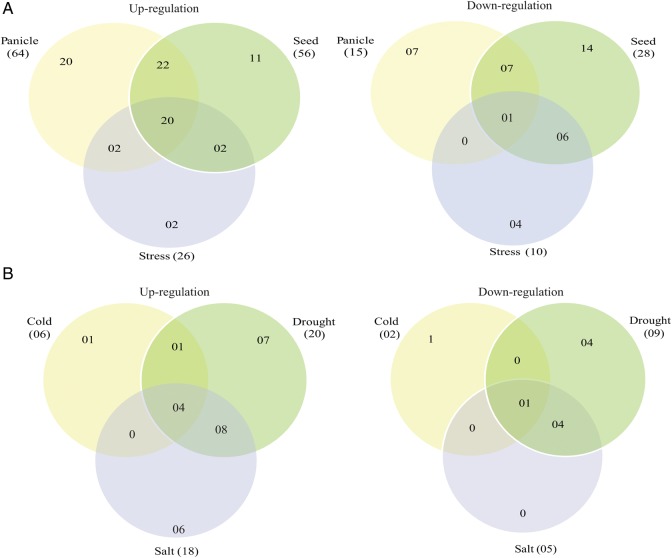
Venn diagram for differentially expressed *OsARMs. OsARM* genes up- and down-regulated in stress and reproductive development showing overlapping expression pattern. Different compartments showing the genes specific to stress, panicle, or seed stage, or involved in stress–panicle, stress–seed, or seed–panicle, or involved in all the three conditions (A). ARM genes up- and down-regulated under different abiotic stress conditions (B). Different compartments showing the genes specific to either one particular stress (salt, drought, or cold), involved in two stresses, or involved in all the three stresses.

The microarray data for *OsARM* genes were generated using Affymetrix rice genome arrays for 7-day-old rice seedlings. Out of 158 genes, probe sets for 14 genes were not available on Affymetrix gene chip array. Unique probe sets were found for 105 genes, 2 for 32 genes, 3 for 6 genes, and 4 for 1 gene. In the case of multiple probe sets, one with the highest baseline expression in the array was chosen for the analysis. Expression profile of 144 *OsARM* genes was analysed under three abiotic stress conditions (salt, cold, and drought) (Figs 3 and 4 and Supplementary Table S5). After defining a criterion of fold change value of ≥2 (either up- or down-regulated) for experimental samples in comparison to control untreated 7-day-old seedlings, 36 *OsARM* genes showed differential expression, with 26 of them being up-regulated and 10 were found to be down-regulated in any of the above given conditions. Four genes (*OsPUB33*, *OsPUB41*, *OsPUB2*, and *OsPUB39*) were found to be up-regulated and a single gene *OsARM14* was found to be down-regulated in all three stress conditions together. Notably, out of 26 up-regulated genes, 7 (*OsPUB8*, *OsARM82*, *OsPUB22*, *OsARM54*, *OsARM11*, *OsPUB43*, and *OsPUB28*) exhibited higher expression under drought stress, whereas 5 genes (*OsARM1*, *OsPUB38*, *OsPUB40*, *OsPUB3*, and *OsPUB46*) were up-regulated under salt stress, specifically. Eight genes (*OsARM7*, *OsPUB30*, *OsPUB32*, *Oryzadillo*, *OsARM8*, *OsARM92*, *OsPUB5*, and *OsPUB6*) were up-regulated under salt and drought stresses together. Whereas, only a single gene (*OsARM13*) was found to be up-regulated under cold and drought stresses together. Interestingly, one gene (*OsPUB28*) was found to be up-regulated in drought and down-regulated under cold stress simultaneously.

Out of the 10 genes, which were down-regulated under drought stress, exclusive down-regulation was observed among four genes (*OsPUB44*, *OsPUB9*, *OsARM59*, and *OsPUB18*). Again, four genes (*OsARM46*, *OsPUB21*, *OsPUB75*, and *OsARM87*) were found to exhibit down-regulation together under drought and salt stress. Besides, a single gene (*OsPUB28*) was found to be specifically down-regulated under cold stress. Expression analysis of 14 *OsARM* genes, which could not be represented on the Affymetrix rice gene chip, was generated through MPSS. Many-fold up-regulation was observed for two genes (*OsPUB45* and *OsARM96)* under all three abiotic stress conditions (Supple-mentary Table S6).

### 3.5. Expression analysis of OsARM genes during development

**Figure 5.**
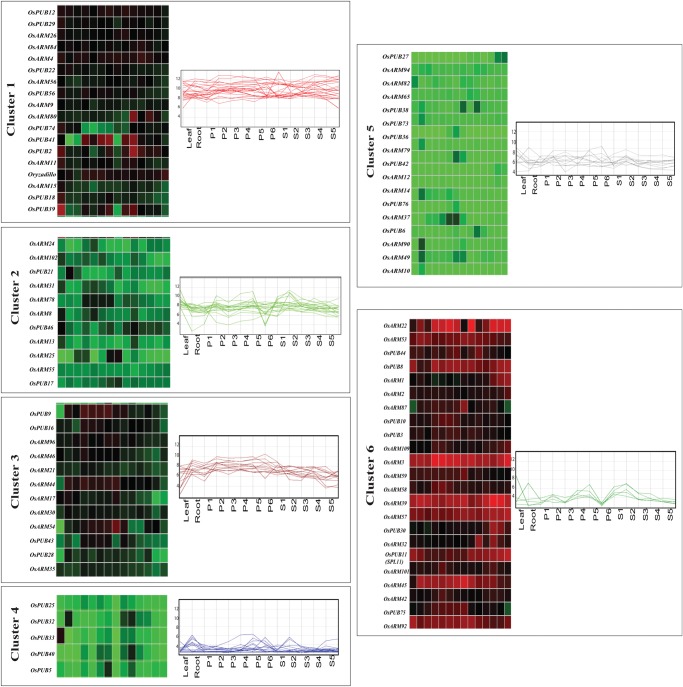
Gene expression profile of *OsARM* genes differentially expressed under developmental conditions using *K-means* clustering tool. Gene expression patterns of 87 (probe sets were not available for two, only 85 could be represented) differentially expressed genes in 11 stages of vegetative and reproductive development categorized into 6 groups using the K-means clustering tool. The normalized log-transformed signal values were plotted for each of the five stages. The Locus ID of genes in the clusters is indicated on the left side of the heatmap.

The expression profile of *OsARM* genes during different stages of development was generated by microarray expression data obtained from Affymetrix rice whole-genome arrays. Corresponding probe sets for 144 *OsARM* genes were found on Affymetrix gene chip; hence, their expression profile could be analysed. For expression analysis during reproductive development, six panicle stages (P1–P6) and five seed (S1–S5) developmental stages were compared with three combined vegetative developmental stages, namely mature leaf, root, and seedling. Altogether, 89 *OsARM* genes were found to be expressing differentially (with fold change ≥ 2), during vegetative and reproductive developmental stages (Figs 3 and 4 and Supplementary Table S7). Cluster analysis was performed for all 89 differentially expressed genes using the K-means clustering algorithm of ArrayAssist (Stratagene). Based on their amplitude of expression, these genes could be clustered into six groups (Fig. 5). Out of these 89 genes, 64 and 55 genes were up-regulated in panicle and seed tissues, respectively. High transcript level was observed commonly for 42 *OsARM* genes in both panicle and seed developmental stages. Exclusive up-regulation was observed for 22 genes during panicle and for 13 genes during seed developmental stages. A total of 15 and 28 genes were down-regulated during panicle and seed developmental stages, respectively. Eight genes (*OsARM10*, *OsPUB74*, *OsPUB35*, *OsARM17*, *OsPUB22*, *OsARM94*, *OsPUB29*, and *OsPUB21*) were commonly down-regulated in panicle and seed stages together. Only 7 genes showed exclusive down-regulation during panicle (*OsARM19*, *OsARM1*, *OsARM2*, *OsARM39*, *OsARM80*, *OsPUB36*, and *OsARM15*) and 20 genes (*OsARM49*, *OsARM90*, *OsARM35*, *OsARM37*, *OsARM92*, *OsARM31*, *OsARM9, OsPUB56*, *OsPUB10*, *OsARM21*, *OsARM96*, *OsARM26*, *OsARM102*, *OsARM53*, *OsPUB44*, *OsARM46*, *OsARM59*, *OsPUB75*, *OsARM14*, and *OsPUB18*) during seed development stages. To understand the relationship between abiotic stresses and plant development, we compared the expression profiles of Armadillo genes during various stages of development and under abiotic stress conditions. A total of 34 genes expressed differentially and exhibited overlapping expression under three abiotic stresses and reproductive developmental stages. Among these, 20 genes (*OsPUB39*, *OsPUB5*, *OsPUB40*, *OsPUB46*, *OsARM13*, *OsPUB28*, *Oryzadillo*, *OsARM8*, *OsARM11*, *OsPUB43*, *OsPUB2*, *OsARM54*, *OsPUB41*, *OsPUB32*, *OsPUB33*, *OsPUB30*, *OsPUB38*, *OsPUB3*, *OsPUB8*, and *OsARM22*) were found to be up-regulated in both developmental stages and stresses, whereas only one gene (*OsPUB21*) was exhibiting down-regulation under these conditions. Besides, a single gene (*OsARM14*), which was showing down-regulation under all three stress conditions and panicle stages at the same time, showed a considerable up-regulation under seed stages. In another instance, expression of (*OsPUB33*, *OsPUB41*, *OsPUB2*, and *OsARM13*) was found to be high and common in all developmental stages and stress conditions. Similarly, transcript levels of *OsPUB28*, which were showing up-regulation under drought stress and down-regulation under cold stress, were found to be high across all developmental conditions.

### 3.6. Validation of microarray expression by real-time Q-PCR

**Figure 6.**
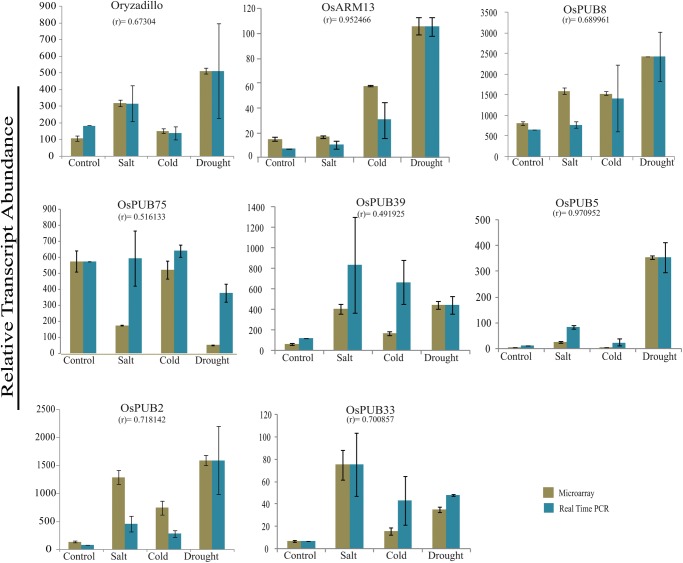
Validation of expression profiles for selected *OsARM* genes in abiotic stress conditions by Q-PCR. Q-PCR and microarray analysis was performed taking two and three biological replicates, respectively. Standard error bars have been shown for data obtained both from microarray and Q-PCR. *Y*-axis represents raw expression values from microarray and normalized expression value from Q-PCR and *X*-axis shows different experimental conditions.

**Figure 7.**
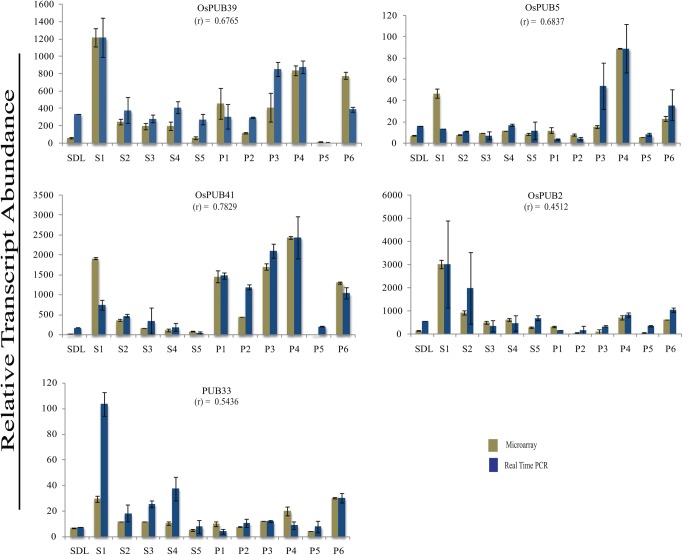
Validation of expression profiles for selected *OsARM* genes in developmental stages by Q-PCR. Q-PCR analysis performed for selected genes to validate their expression profiles during various stages of development. Standard error bars have been shown for data obtained both from microarray and Q-PCR. *Y*-axis represents raw expression values from microarray and normalized expression value from Q-PCR and *X*-axis shows different experimental conditions.SDL, seedling; S1–S5, stages of seed development; P1–P6, stages of panicle development.

Among 36 *OsARM* genes expressing differentially under abiotic stress conditions, expression pattern of eight significant candidate genes was validated experimentally, employing quantitative real-time RT–PCR. All the eight genes showed anticipated expression pattern in most of the conditions and hence, could be correlated with microarray expression pattern. Similarly, differential expression of five genes under developmental stages was validated by Q-RT–PCR analysis. Statistical analysis also confirmed a positive correlation association between the microarray and real-time analysis (Figs 6 and 7 and Supplementary Table S8). However, the magnitude of expression varied in some samples. This kind of variation in expression by these two techniques, i.e. microarray and real-time PCR, has also been observed previously.^33,34^ The real-time PCR efficiency was also calculated and was found to be ∼92%, which is in the acceptable range.

### 3.7. Expression analysis of duplicated genes

**Figure 8.**
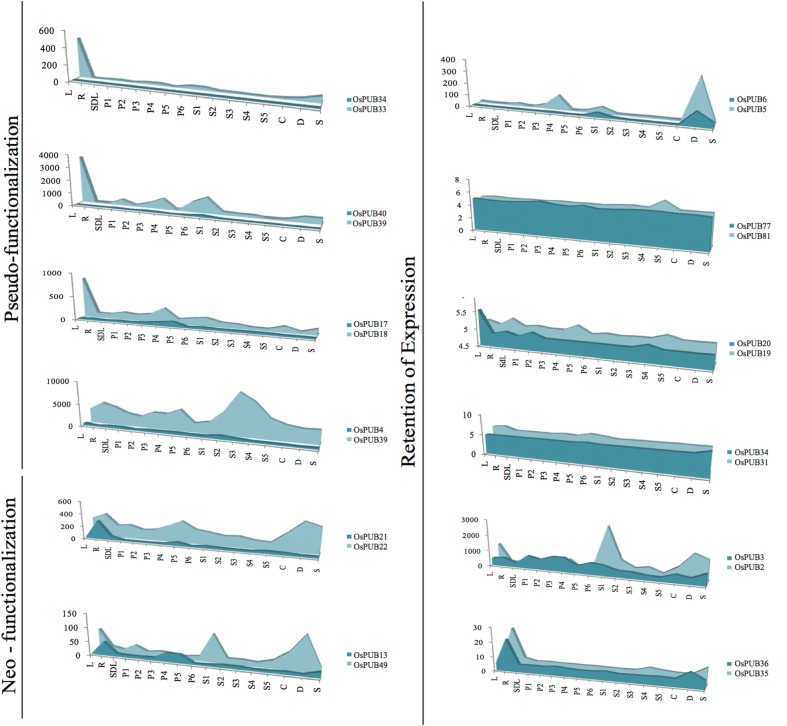
Expression pattern of duplicated *OsARM* genes. The expression values of segmentally and tandemly duplicated genes obtained from microarray data were compared in leaf (L), root (R), and 7-day-old seedling (SDL) tissue, in various stages of panicle development (P1–P6), seed development (S1–S5), and under cold stress (CS), dehydration stress (DS), and salt stress (SS). Each area graph represents compilation of the mean normalized signal intensity values from 17 stages of development/stress conditions. Gene pairs have been grouped into retention of expression, neo-functionalization, and pseudo-functionalization based on their respective profile.

To study the evolution and response of the duplicated *OsARM* genes under abiotic stress and during developmental conditions, their expression profile was generated using microarray data. The signal intensity values for 12 segmentally duplicated genes in three vegetative stages, six panicle, five seed, and three abiotic stress conditions are represented as an area chart (Fig. 8). The expression analysis of six pairs of segmentally duplicated paralogous genes (*OsPUB31–OsPUB34*, *OsPUB2–OsPUB3*, *OsPUB35–OsPUB36*, *OsARM77–OsARM81*, *OsPUB5–OsPUB6*, and *OsPUB19–OsPUB20*) shows identical expression pattern throughout the range of stress and developmental conditions and thus shows retention of expression. However, the level of expression varied between these duplicated genes. Four pairs of duplicated genes (*OsPUB33–OsPUB34*, *OsPUB39–OsPUB40*, *OsARM4–OsARM39*, and *OsPUB17–OsPUB18*) exhibited pseudo-functionalization, due to negligible expression shown by one of the duplicated paired partners. The acquisition of new function and hence, neo-functionalization has been shown by two pairs of segmentally duplicated gene (*OsARM13–OsARM49* and *OsPUB21–OsPUB22*) where both the duplicated partners display clearly distinguishable expression profile.

### 3.8. Cis-regulatory promoter elements of stress-inducible genes

*In silico* analysis of 1 kb upstream region (from translation start site) of the *OsARM* genes, which were differentially expressed under abiotic stresses revealed the presence of various regulatory elements, such as ABRE (Abscisic acid Responsive Element), ARE (Anaerobic Response Element), LTR (Low Temperature Res-ponsive), MBS (Myb-Binding Site), HSE (Heat Shock Element), GCN4 (endosperm expression), and TC-rich repeats (defence and stress response). These motifs have been previously known to regulate various stress and plant developmental responses.^35,36^ These motifs are present variably in the promoter of selected stress-induced *OsARM* genes. Most regulatory elements are present in the 1 kb upstream region of *OsPUB2*, which was commonly up-regulated in all the three abiotic stresses, with eight elements followed by *OsPUB75* (down-regulated in salt and drought) with seven regulatory elements. All the *OsARM* genes included in the analysis contained at least four regulatory elements in their promoter region. A detailed analysis confirmed the relationship of gene response and presence of corresponding *cis*-element in their promoter region (Supplementary Fig. S2 and Supple-mentary Table S8).

## 4. Discussion

### 4.1. Organization of Armadillo genes in rice genome

ARM repeat proteins display ubiquitous presence across eukaryotes and expanded rather dramatically in the land plant lineage. In the present study, extensive database searches facilitated identification of 158 Armadillo repeat proteins in rice genome. To identify orthologous clades among ARM proteins in rice and *Arabidopsis*, a combined phylogenetic tree was generated. Due to the presence of inconsistent number of ARM repeats and high degree of sequence divergence between them, it was difficult to deduce phylogenetic relationship between these proteins, which has also been observed previously.^14^ Assuming all ARM repeat proteins were originally derived from a common ancestor; the full-length protein sequence was used for phylogeny prediction. The phylogenetic analysis revealed the distribution of ARM proteins into 15 major clades. It was observed that the nodes at the base of the major groups were not well supported (low bootstrap score), but nodes at the base of many minor groups were robust (bootstrap values > 90%). A large number of ARM proteins from both rice and *Arabidopsis* were falling into the same subgroups based on their related domain composition, suggesting a common ancestry of ARMs in two diverse species. We also observed that 39 of 158 OsARM proteins outlined a monophyletic group (Group 15, Fig. 1) but no *Arabidopsis* specific subgroup was identified. The family size difference between *Arabidopsis* and rice may be the consequence of differential expansion among subfamilies. We also analysed domain contents and organizations of predicted OsARM repeat proteins. Besides ARM, there are various other accessory domains present in many proteins. As expected, proteins with similar domain organizations tend to be clustered together based on overall sequence homology. It was observed that 47 rice *PUB/ARM* proteins together with *Arabidopsis* orthologues are majorly dispersed in Groups 1–4 based on the kind and number of ARM repeats they possess. To examine the relationship among the large number of identified U-box/ARM proteins in *Arabidopsis* (41) and *Oryza* (47), the phylogenetic tree was constructed using complete protein sequences. The U-box proteins were divided into five distinct phylogenetic groups with 85–99% bootstrap support (Supplementary Fig. S1). Mudgil *et al.*^14^ in their study have predicted a unique N-terminal region in U-box, termed as UND domain in 17 of the U-box/ARM proteins in *Arabidopsis*. The ATPUB/ARM proteins having UND region were found to be closely coinciding with 17 OsPUB/ARM proteins. Sequence analysis also confirmed the presence of UND-like domain in these OsPUB proteins. Moreover, the number and arrangement of ARM repeats in closely associated PUB proteins in the phylogenetic map was also found to be identical. The division of PUBs in different groups indicates the evolutionary path taken up by these proteins due to the proliferation of ARM repeats within them. On the other hand, the phylogenetic comparison using only U-box domain sequence suggests an independent evolution of this domain in both species (data not shown). The similarity between wide range of domains and motifs found in the *Arabidopsis* and rice Armadillo members suggests that the domain/motif arrangement of most of the members of Armadillo family was established before the divergence of rice and *Arabidopsis.* In general, except U-box/ARM, which are interestingly extended in four different groups in the phylogenetic tree (Groups 1–4), HECT (Group 5), IBB (Group 6), C2/ARM (Group 7) proteins, the majority of *OsARM* genes do not show a distinct clade-wise grouping and many of the members did not fall in any of the defined clade. In agreement with previous studies, indicating conservation of the *Arabidopsis Arabidillo-1*, *Arabidillo-2* genes across the land plants, which are involved in root architecture development, were found to be having a single orthologue in rice as *Oryzadillo.*^37–39^ Similar conservation was observed in the case of proteins containing distinguished domains such as PUB, C2 (Ca^2+^ binding motif), ARK (ARM repeat-containing kinesin), and Importin-α/ARM proteins between rice and *Arabidopsis*. Perhaps, the association of ARM repeats with several other functional domains might be allowing them to interact with various unrelated proteins.

### 4.2. Potential role of OsARMs in abiotic stress and plant development

Our microarray expression analysis of *OsARM* gene family confirmed the presence of some unique ARM repeat proteins, which might play significant role under abiotic stress and in plant development (Supple-mentary Tables S5 and S7). The expression analysis showed that a subset of *OsARM* genes was expressing differentially under abiotic stress conditions. Among these 36 differentially expressed genes, 21 consists a U-box domain. Specifically, in the plant system, regulated protein degradation has been implicated in a number of pathways as diverse as growth and development, light and hormonal signalling, embryogenesis, leaf senescence, and biotic and abiotic stress.^40–45^ This association of large number of U-box proteins with ARM repeats suggests a significant role of these repeats in protein degradation and key regulatory pathways, including stress signalling. This statement was further supported by the fact that several *Arabidopsis* ARM/U-box proteins were also confirmed to be expressing in different tissues under varying growth conditions.^16^ Furthermore, it may be speculated that the ARM repeats may mediate interactions with large number of proteins, conferring substrate versatility to the proteasomal degradation pathway.

The overall up-regulation under abiotic stress and developmental conditions has been observed in *OsPUB33*, whereas its orthologue *ATPUB23* has been established to involve in response to water stress and as a negative regulator of PAMP-triggered immunity.^46,47^Another *OsARM* gene (*OsPUB5*) that showed up-regulation under drought and salt stress conditions has its functionally characterized orthologue in *Arabidopsi*s (*At1g10560*) reported to be involved in plant ABA response.^44^ Further, sole representative of LRR/ARM/F-box domain containing *ARABIDILLO-1*,*-2*,*Oryzadillo* was also found to be many-fold up-regulated under salt and drought conditions. Recent finding suggests that homologues of these proteins are conserved across plants species exhibiting novel and overlapping functions.^37,38^ Interestingly, some of the *OsARMs* (*OsPUB28*, *OsARM14*, *OsPUB75*) were found to be showing elevated expression under developmental conditions but a significant down-regulation under stress conditions. Another U-box/ARM gene (*OsPUB6)*, which was found to be highly, expressed under drought and salt stress conditions in the microarray analysis, share sequence similarity with *Arabidopsis AtPUB18.* In *Arabidopsis*, the role of *PUB18* has been established in salt and ABA-mediated drought stress response.^13^ A different *OsPUB/ARM* (*OsPUB8)*, found to be induced under drought stress, reproductive, and developmental conditions, is an orthologue of *Arabidopsis ATPUB9*, which regulates plant ABA response.^48^ Similarity in the expression pattern among orthologous group of genes suggests that closely related genes are not evolving separately and can be represented as functional orthologue both in rice (monocot) and in *Arabidopsis* (dicot).

Furthermore, two of the *OsARM* family genes (*OsARM45* and *OsARM87)* harbour C2 domain, specifically known to be involved in the calcium signalling pathways.^49^ In the expression analysis, *OsARM87* was found to be showing significant down-regulation under drought and salt stress conditions; however, *OsARM45* did not show differential expression under any of the conditions but might be involved in the interaction with microtubules, since a cytoskeletal role has been predicted for its orthologue *ATCSI1/C2* in *Arabidopsis.*^50^ This can also be speculated that the ARM/C2 proteins possibly be involved in the interaction with the target proteins, thereby localizing them to the membrane. Further, Importin-α/ARM proteins are known to aid in localizing cNLS (classical Nuclear Localization Signal) containing proteins from cytoplasm into the nucleus.^51^ Expression analysis showed down-regulation of IBB-domain containing (*OsARM2*) gene in the panicle and up-regulation in seed stages, although it was not found to be expressed under any of the stress conditions. In *Arabidopsis*, ARM/BTB/POZ domain containing protein ARIA (ARM repeat protein interacting with ABF2) has been implicated in ABA-dependent gene expression.^7^ Whereas in rice, the transcript levels of *ARIA* orthologue (*OsARM42*) were found significantly high under all developmental conditions, but no stress-responsive expression was found. Apart from abiotic stress-regulated genes, a large subset of ARM proteins (89) were found to be differentially regulated under developmental stages (Supple-mentary Table S7). Many of these showed preferential accumulation of transcripts in given developmental stages. The comparison of specific regulation of these genes under vegetative and seed developmental stages has allowed segregation of transcripts in six co-expression groups (Fig. 5). The number of genes varied from 5 (cluster IV) to 23 (cluster VI) in these clusters. Cluster I includes 18 ARM genes, which are highly expressed, and cluster V includes 5 low expressing ARM genes in all the developmental stages analysed. Eleven genes in cluster II also exhibited lower expression in all the conditions analysed. The possible explanation for low expressing *OsARM* genes in various developmental stages analysed might be due to their expression in specific cell-type(s) or tissue/condition other than those included in this study. However, other ARM genes exhibited transcript abundance in one or more distinct developmental stages analysed. Twelve genes in cluster III were expressed preferentially in stages of panicle development and their expression was lower in seed development stages. Eleven ARM genes in cluster II were preferentially expressing during P3, P4 and S1, S2 stages, but their expression was restricted, specifically during P5 stages. These results suggest the involvement of ARMs in various stress and developmental events in rice.

Furthermore, we found that six pairs of segmentally duplicated genes were also expressing differentially under abiotic stress conditions (Supplementary Table S10). Although, five of these duplicated pairs were showing similar expression under the same stress conditions, one pair (*OsPUB21–OsPUB22*) showed highly diverged expression patterns as the former was up-regulated under drought and the latter was down-regulated in the same condition. For *OsPUB45*, expression was analysed in MPSS and it was found to be up-regulated under stress conditions. It has been found that the independent individual ARM repeats are short of definite structure and function; however, the presence of multiple repeats in tandem provides a definite structural and functional composure to the repeat proteins, which are mostly associated with some other functional domain.^11^ Nevertheless, our expression analysis revealed many ARM repeat proteins without any associated domain to be differentially regulated under stress conditions, which might indicate a plant-specific functional role for them during stress and developmental conditions.

### 4.3. Cis-regulatory elements in the promoter of OsARM genes

We could identify several stress-responsive motifs in the promoter region of abiotic stress-inducible *OsARM* genes. *Cis*-acting elements have been previously known to control the molecular processes of various plant stress and developmental responses.^52–56^ For example, in *OsARM13* and *OsPUB2*, which were found to be highly expressed under cold and drought stresses, motifs such as ABRE, HSE, MBS, and LTR were found to be present variably. Interestingly, the promoter region of the *OsPUB75* gene, which has been down-regulated under seed stages, stress, and drought conditions, contains several stress and developmental-related *cis*-elements. Sequence elements such as GCN4 and SKN-1 essential for endosperm-specific expression were identified at multiple locations in the 1 kb upstream regions of seven genes. Transcript level of these genes under developmental conditions was also found to be many-fold high in the expression analysis. Further, TC-rich repeat responsible for defence and stress responses have been found in the promoter region of three genes, suggesting their possible role during these conditions.

### 4.4. Duplication events contributed to the proliferation and functional diversification of OsARMs in rice

A number of *OsARM* genes were found to be duplicated either segmentally (∼10%) or in tandem (∼19%), suggesting a role of gene duplication in the expansion and evolution of this gene family in rice genome. Gene duplication serves as a mechanism to increase functional diversity. Duplication events often lead to diversification of gene function such as neo-functionalization or pseudo-functionalization.^57,58^ To know the functional redundancy/diversification of duplicated ARM genes, the expression profiles were generated for 12 pairs of duplicated genes, covering the spectrum of abiotic stresses as well as vegetative and reproductive developmental stages (Fig. 8).

Here, parallel retention (retention of expression) of a stress response in six pairs of duplicated genes with some quantitative differences indicates that they maintain similar stress response as their ancestor. One possible explanation for this high rate of retention is that the duplicate genes we analysed tend to be derived from more recent duplication events. Since there is a direct relationship between the degree of expression divergence of duplicated genes and their divergence times,^59^ the degree of stress-response change is most likely correlated with the age of gene duplication events as well. The relative frequency analysis of paralogous genes using synonymous substitution rate *Ks* showed that younger duplicate genes were more likely to be retained during stress responses and thus exhibits retention of expression. Intriguingly, we found that the gene pair (*OsARM13–OsARM49*) with the highest *Ks* rate (1.15) has been predicted to be the earliest diverging (∼88.65 MYA) followed by (*OsPUB21–OsPUB22*) with the *Ks* rate (0.813) and diverging (∼62.56 MYA) exhibited neo-functionalization. Hence, the elevated rate of duplication and retention of stress responses by these genes in ARM family suggests that they might be crucial for responses to stress. The *Ka/Ks* analysis showed that the Armadillo repeats are undergoing purifying selection in both species, as all the segmentally duplicated Armadillo pairs from *Arabidopsis* and rice showed *Ka/Ks* < 1.

## 5. Conclusion

The present study provides a comprehensive account of the Armadillo gene family in rice. Protein sequence and phylogenetic analysis revealed the evolutionary conservation of this group of proteins across different plant species. Several developmental stage-specific, abiotic stress-responsive *ARM* genes belonging to various groups have been identified. The results further encourage stress and developmental-specific functional analysis of a few members of this gene family in crop plant rice and their further characterization for agricultural importance.

## Supplementary data

Supplementary Data are available at www.dnaresearch.oxfordjournals.org.

## Funding

This work is partially supported by internal grants of University of Delhi and Department of Biotechnology (DBT) India to GKP. M.S acknowledge University Grants Commission (UGC), India for fellowship. AS, VB and AlS acknowledge Council of Scientific Research (CSIR) for their fellowship.

## Supplementary Material

Supplementary Data
