# Supplementary material for: Comprehensive Expression Analysis of Rice Armadillo Gene Family During Abiotic Stress and Development
Source: DNA Res. 2014 Jan 6;21(3):267–83. doi: 10.1093/dnares/dst056 (PMC4060948; doi:10.1093/dnares/dst056)
Supplement: Supplementary Data [file supp_21_3_267__index.html]

Comprehensive Expression Analysis of Rice Armadillo Gene Family During Abiotic Stress and Development — Comprehensive Expression Analysis of Rice Armadillo Gene Family During Abiotic Stress and Development — Supplementary Data 

# Comprehensive Expression Analysis of Rice Armadillo Gene Family During Abiotic Stress and Development

## Supplementary Data

Supplementary Data

**Files in this Data Supplement:**

- Supplementary Data - Doc file
- Supplementary Figure 1 - tif file
- Supplementary Figure 2 - tif file
- Supplementary Table 1 - xlsx file
- Supplementary Table 2 - xlsx file
- Supplementary Table 3 - xlsx file
- Supplementary Table 4 - xlsx file
- Supplementary Table 5 - xlsx file
- Supplementary Table 6 - xlsx file
- Supplementary Table 7 - xlsx file
- Supplementary Table 8 - xlsx file
- Supplementary Table 9 - xlsx file
- Supplementary Table 10 - xlsx file
- Supplementary Table 11 - xlsx file
