# Supplementary material for: Comprehensive Expression Analysis of Rice Armadillo Gene Family During Abiotic Stress and Development
Source: DNA Res. 2014 Jan 6;21(3):267–83. doi: 10.1093/dnares/dst056 (PMC4060948; doi:10.1093/dnares/dst056)
Supplement: Supplementary Data [file supp_dst056_dst056supp.doc]

**Figure S1. Promoter analysis of differentially regulated *OsARM* genes.**

*Cis*-regulatory element in the 1kb upstream region (from translation start site) of the promoter of *OsARM* genes were analyzed *in-silico*. The elements present in the forward strand and reverse strands are indicated by the triangle mark above and below the line, respectively. Induction of the genes in different abiotic stresses is indicated on the right side of each gene. All the *cis*- elements and their positions are listed in Table S9.

**Figure S2. Phylogenetic relationship between *Arabidopsis* and rice U-box genes.**

A phylogeny created with the full-length protein sequences of PUB-ARMs in *Arabidopsis* and rice. The alignment of full-length protein sequences of rice Armadillo genes was done using ClustalX 2.0.12. An unrooted neighbor-joining (NJ) tree was generated using the p-distance substitution model in MEGA 5. Bootstrap analysis was performed with 1000 replicates to obtain a support value for each branch.

**Table S1** Gene attributes for 158 predicted *OsARM* genes.

(XLSX)

**Table S2** Segmental and Tandem Duplication in *OsARMs.*

(XLSX)

**Table S3** Segmental and Tandem Duplication in *AtARMs.*

(XLSX)

**Table S4** Estimation of the age of segmentally duplicated Armadillo genes in *A. thaliana, O. sativa.*

(XLSX)

**Table S5** Microarray expression data for *OsARMs* under three abiotic stress conditions.

(XLSX)

**Table S6** MPSS data for 14 *OsARM* genes, which could not be represented on the Affymetrix gene chip.

(XLSX)

**Table S7** Microarray expression data for *OsARMs* during development.

(XLSX)

**Table S8** List of primers used for real time PCR expression analysis of Os*ARMs.*

(XLSX)

**Table S9** *Cis*-regulatory element analysis of differentially expressed *OsARMs* using PlantCARE database.

(XLSX)

**Table S10** Microarray expression values for segmental duplicated *OsARM* genes.

(XLSX)

**Table S11** Microarray expression absolute values for stress and developmental conditions. (XLSX)
