# Supplementary figures and images for: Comprehensive Expression Analysis of Rice Armadillo Gene Family During Abiotic Stress and Development
Source: DNA Res. 2014 Jan 6;21(3):267–83. doi: 10.1093/dnares/dst056 (PMC4060948; doi:10.1093/dnares/dst056)

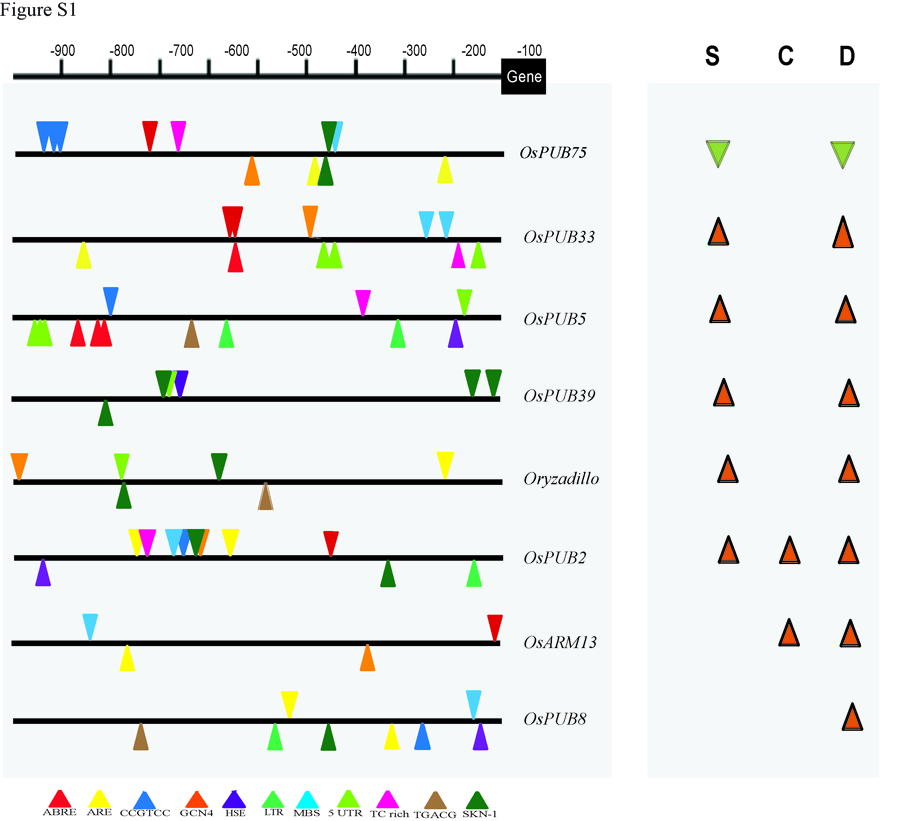

Supplement: Supplementary Data [file supp_dst056_dst056supp_fig1.tif]

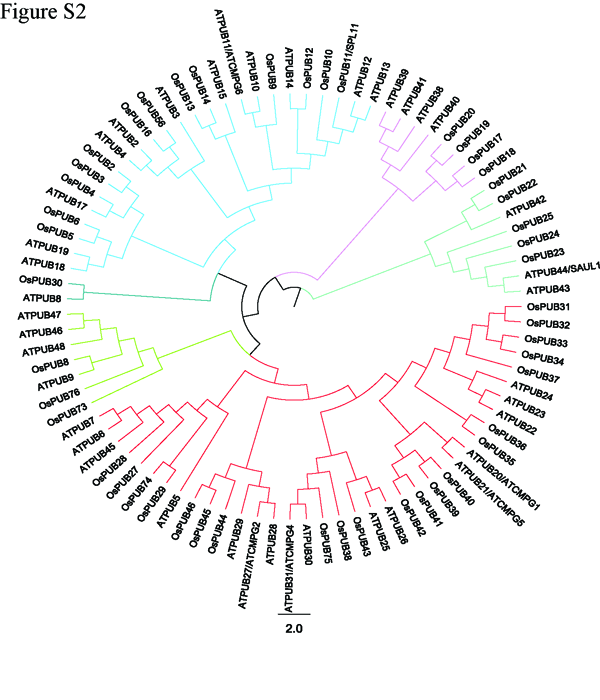

Supplement: Supplementary Data [file supp_dst056_dst056supp_fig2.tif]
